# Mutations in the voltage-gated sodium channel associated with pyrethroid resistance in *Aedes albopictus* in Yucatan, Mexico

**DOI:** 10.1590/S1678-9946202466062

**Published:** 2024-11-11

**Authors:** Wilbert Antonio Chi-Chim, Julian Everardo Garcia-Rejon, Julio Cesar Tzuc-Dzul, Lourdes Talavera-Aguilar, Rosa Cetina-Trejo, Nohemi Cigarroa-Toledo, Viviana Caamal-Villanueva, Diana Guadalupe Argaez-Sierra, Carlos Marcial Baak-Baak

**Affiliations:** 1 Universidad Autónoma de Yucatán, Centro de Investigaciones Regionales "Dr. Hideyo Noguchi", Laboratorio de Arbovirología, Mérida, Yucatán, Mexico; 2 Universidad Autónoma de Yucatán, Centro de Investigaciones Regionales "Dr. Hideyo Noguchi", Laboratorio de Biología Celular, Mérida, Yucatán, México; 3 Universidad Autónoma de Yucatán, Facultad de Química, Mérida, Yucatán, Mexico

**Keywords:** Asian tiger mosquito, Non-synonymous mutation, Wild homozygous, Insecticide resistance

## Abstract

*Aedes albopictus* (Skuse) is a competent vector of dengue and Zika viruses in Mexico. Monitoring the level of resistance of local population is essential due to its epidemiological significance. This study aimed to identify mutations in the voltage-gated sodium channel (VGSC) as one of the mechanisms responsible for pyrethroid insecticide resistance in *Ae. albopictus*. Immature samples were collected in a rural town in Yucatan, Mexico, from May to October 2021. The pyrethroid insecticide lambda-cyhalothrin was impregnated in CDC bottles and bioassays were conducted on *Ae. albopictus* populations 3–5 days after emergence. The mosquitoes were susceptible to the insecticide. Females were taken for total DNA extraction. Fragments of domains II, III, and IV of the voltage-gated sodium channel (VGSC) were amplified and sequenced. The presence of synonymous and non-synonymous mutations was found in positions 1532 and 1534 of domain III of the sodium channel gene (VGSC). No mutant alleles in domain IV were detected. A homozygous mutant (ACG) coding for the amino acid threonine (1008Thr) was identified in domain II. Domain III included three heterozygous alleles (P1528S, L1530S, and Ile1410Thr). This last heterozygous allele is reported for the first time in Mexico. Homozygous mutants encoding the amino acids serine/serine and serine/proline in domain III were observed. These have been reported in *Aedes aegypti* from Mexico, but not yet in *Ae. albopictus*. This represents new findings for the region, as *Ae. albopictus* has only been introduced there for approximately five years. In conclusion, non-synonymous mutations were found in *Ae. albopictus* in a rural area of Yucatan, emphasizing the importance of integrated vector control to prevent Asian tiger mosquitoes from spreading these resistant alleles.

## INTRODUCTION

*Aedes albopictus* (Skuse), commonly known as the Asian tiger mosquito, is classified as one of the invasive vectors worldwide. The species exhibits significant genetic and physiological adaptability, enabling it to successfully colonize many environments^1,2^. Moreover, it demonstrates a high degree of effectiveness in participating in the transmission cycles of endemic infections within the invaded region^3-5^. In the absence of humans, the Asian tiger mosquito can feed on mammals and avian species^2,6,7^. The wide range of hosts incorporated into its diet endows it with the capacity to transmit many pathogens. In America, scientists have identified eight arboviruses that have been isolated from *Ae. albopictus*, however, experimental transmission has been demonstrated for a total of 16 arboviruses^2^. In Mexico, it is a competent vector for dengue and Zika viruses^3,5^.

Dengue is the most important arbovirus in Mexico owing to its morbidity and mortality rates^8^. Pyrethroids such as lambda-cyhalothrin are chemicals used to kill the dengue virus’ major vector, *Aedes aegypti*. Over an extended period, these chemical compounds were used on *Ae. aegypti* and *Culex quinquefasciatus*, resulting in alterations in the voltage-gated sodium channel (VGSC) associated with the development of resistance to pyrethroids^9,10^. The frequent emergence of pyrethroid "knockdown" resistance (kdr) can be attributed to the presence of non-synonymous mutations in the transmembrane protein of the voltage-gated sodium channel (VGSC), resulting in a reduced binding affinity for pyrethroids^11,12^. Given *Ae. albopictus* occupies comparable ecological niches to *Ae. aegypti*, it is imperative to periodically evaluate the susceptibility and resistance status of mosquito populations to pyrethroids insecticides^2^. The Asian tiger mosquito was first identified in Mexico in 1988. Currently, *Ae. albopictus* has been documented in 21 states of Mexico during a span of 35 years, including Yucatan State^13^. Despite its widespread distribution and potential as a vector of arboviruses in Mexico, research examining the susceptibility profile of this particular species are still scarce, and the genotyping of kdr mutations has not been consistently conducted^11,12,14,15^. The aim was to identify the presence of mutations in the voltage-gated sodium channel (VGSC) as a mechanism associated with pyrethroid insecticide resistance in *Ae. albopictus*.

## MATERIAL AND METHODS

### Study site and collection of immature stages

A comprehensive entomological study was conducted in the Polaban site (20°44’0" N, 89°13’6" W) from May to October 2021. Polaban is a rural village situated 62 kilometers from Merida city, the capital of Yucatan State, Mexico. The survey involved the collection of larvae and pupae. Immature mosquitoes originating from both artificial and natural breeding sites were collected with nets, turkey basters, and pipettes. These specimens were subsequently transferred into plastic containers, which were appropriately labeled with the date of collection, study site, and unique sample identification number^10^. The immature stages were then transported to the Laboratorio de Arbovirologia at the Universidad Autonoma de Yucatan. The larvae were raised until adulthood. The mosquitoes identified as *Ae. albopictus* ^16^ were selected and reproduced in the insectary to yield eggs. The adult mosquitoes were placed into enclosed entomological cages with screens (30cm^3^) for colony maintenance. They were then given a meal consisting of sucrose solution (10%), delivered using cotton pads^10^. Adult females were blood-fed on anesthetized mice (Animal Ethic Approval: EI082015-CIR-UADY). Eggs collected on filter paper were induced to hatch, and the resulting larvae were reared until they emerged as adults (F1). Larvae were placed in plastic trays measuring 57×40×30 cm and filled with 5 L of tap water and 1 g of goldfish food (Biopro^®^, Monterrey, NL, Mexico). Food supplement was added every two days. The growth of immature mosquitoes was sustained under controlled conditions of 28±1 °C, 80% relative humidity, and a 12-hour light-dark cycle. Insecticide susceptibility bioassays were conducted using emerging female adults from the parental generation (F1) from established colonies in the insectary.

### Insecticide susceptibility test in *Ae. albopictus*

The evaluation of *Aedes albopictus*’ susceptibility to pyrethroid insecticide was conducted using the CDC bottle (Wheaton 250 ml) bioassay procedure. Female mosquitoes aged from 3 to 5 days and unfed were exposed to the chemical lambda-cyhalothrin (Fogger 7 CE^®^). Using a mouth aspirator, males and females were separated and 15 females were placed in each bottle. The experiment was repeated four times following the diagnostic dose recommended for *Aedes* mosquitoes established by the Centers for Disease Control and Prevention (CDC) and other works^11,15^, which specified 10 µg per bottle. The knockdown effect was measured at regular intervals of 15min until the designated diagnostic time of 2h. The mortality rate was documented within 24h after exposure according to the results interpretation and the criteria proposed by the World Health Organization (WHO). A mortality rate from 98 to 100% indicates the population is susceptible to the insecticide. On the other hand, a mortality rate from 80 to 97% suggests possible resistance and must be confirmed. Finally, a mortality rate of less than 80% indicates resistance^15^.

### Extraction of genomic DNA and detection of kdr alleles in *Ae. albopictus*

Genomic DNA was extracted from *Ae. albopictus* females individually using the salt extraction technique^17^. The extracted DNA was stored at −20 °C for posterior use. The identification of kdr alleles of the voltage-gated sodium channel gene in *Ae. albopictus* was performed using oligonucleotide sequences that amplify three transmembrane domains (II, III, and IV). We used three primer sets to amplify domain segments: aegSCF20 (gacaatgtggatcgcttccc) and aegSCR21 (gcaatctggcttgttaacttg) for Domain II; aegSCF7 (gagaactcgccgatgaactt) and aegSCR7 (gacgacgaaatcgaacaggt) for Domain III, albSCF6 (tcgagaagtacttcgtgtcg) and albSCR8 (aacagcaggatcatgctctg) for Domain IV^18^. Molecular polymerase chain reaction (PCR) assays were performed in a volume of 25 μL for each reaction, amplifying three domains of the sodium channel gene, using the following quantities for each reaction: 2.3 μL reaction buffer (5x); 2.0 μL of MgCl_2_ (25 μM); 0.2 μL of dNTP’S (10 μM); 0.5 μL of specific primers (10 μM); 5 U/ μL of Taq polymerase (ThermoFisher Scientific); and 2.5 μL of genomic DNA sample. Denaturation conditions were at 94 °C for 2 min, followed by 35 cycles of 94 °C for 30 s, 60 °C for 30 s, and 72 °C for 30 s, with a final extension of 72 °C for 8 min^9,18^. The amplicons were observed on 2% agarose gels containing 0.5 µg/mL ethidium bromide, using a Doc™ XR+ Gel Documentation System. The PCR products were purified using the Zymoclean DNA recovery kit Cat (Zymo Research, Irvine, CA, USA), and subsequent sequencing was performed using a 3500xL DNA sequencer (Applied Biosystems, Foster City, CA, USA). The sequences were analyzed and edited using the Bioedit (version 7.2.5, Tom Hall, North Carolina, USA) and Chromas programs (version 2.6.6, Technelysium Pty Ltd, Brisbane, Australia). The CodonCode Aligner (version 11.0.2, CodonCode Corporation, Massachusett, USA) program was employed to analyze mutations and kdr alleles. The *Ae. albopictus* genome (GenBank accession Nº MH84955–MH384958 and MH384950–MH384961) was used as a template to identify mutations.

## RESULTS

**Figure 1:**
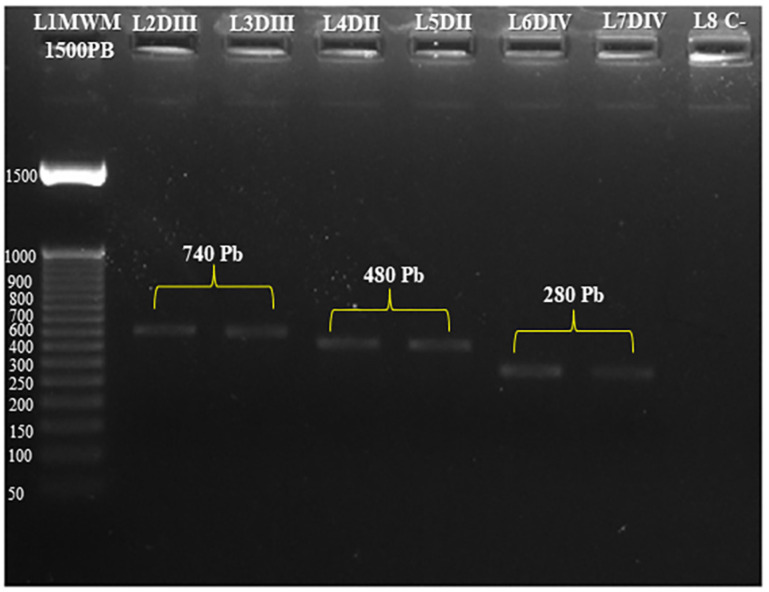
Amplification of partial sequences of the voltage-gated sodium channel (VGSC) domains in *Ae. albopictus*, the target of action of pyrethroid insecticides. LI MWM: 1500 bp molecular weight marker (ThermoFisher Scientific); L2DIII and L3DIII: partial amplified segment of Domain III (740 bp); L4DII and L5DII: partial amplified segment of Domain II (480 bp); L6DIV and L7DIV: partial amplified segment of Domain IV (280 bp); and L8C-: negative control.

The study findings indicated that the rural population of *Ae. albopictus* exhibited susceptibility to lambda-cyhalothrin. The percentage of knockdowns observed exceeded 98% within a 15-min period of exposure. The mortality rate reached 100% within a 24-h period. Genomic DNA was extracted from 50 *Ae. albopictus* females exposed to the insecticide lambda-cyhalothrin. Figure 1 displays the amplified region of the sodium channel domains.

Two *Ae. albopictus* females were subjected to sequencing analysis. The sequences were submitted to GenBank and assigned the accession numbers OR877955 (Domain II), OR877956 (Domain III), OR877957 (Domain IV), OR877958 (Domain III), and OR877959 (Domain IV). The sequences produced in this study were aligned and compared with sequences that are accessible in the GenBank database. For the Domain segment II, the sequences with accessions number MK201608, AB828338, AB827810, and LC485547 were compared. For Domain segment III, the sequences with accessions numbers MK201621, MK201631, and MH384958 were compared. Table 1 presents the identification of synonymous mutations that have unknown activities in protein structure.

**Table 1:** Amino acid substitution identified within a 480bp partial segment of domain II and within a 740bp partial fragment of Domain III of the *Aedes albopictus* voltage-gated sodium channel. Bold letters indicate amino acid substitutions associated with pyrethroid insecticide resistance reported in other mosquitoes’ species that were identified in our study with *Ae. albopictus* ^18,19,21,23,24^.

Domain	Characteristic	Codon change	Amino acids
II	Homozygous	2931T>G	Ile977Arg
II	Homozygous	2940G>A	Arg980Gln
II	Homozygous	2983G>C	Ala995Leu
II	Homozygous	2983A>T	Tyr995Phe
**II**	**Homozygous**	**2994G>T**	**Asp998Tyr**
**II**	**Homozygous**	**2994C>T**	**Asp998Asp**
II	Homozygous	3009C>A	Thr1003Pro
II	Homozygous	3024T>A	Thr1008Phe
II	Homozygous	3024T>A	Thr1008Ser
II	Homozygous	3024A>G	Thr1008Thr
**II**	**Heterozygous**	**3042T>C**	**Leu1014Phe**
**II**	**Homozygous**	3053T>A	Ile1018As
**II**	**Heterozygous**	**3057T>C**	**Leu1019Ser**
II	Heterozygous	3150C>T	Ala1050Val
II	Homozygous	3177C>T	Ser1059Ser
**II**	**Homozygous**	**3186T>C**	**Ser1062Pro**
II	Homozygous	3189T>G	Ser1063Ala
II	Homozygous	3282A>T	Ser1094Cys
**II**	**Homozygous**	**3243G>A**	**Ala1081Ser**
III	Homozygous	4442A>G	Asn1481Asp
**III**	**Homozygous**	**4470C>T**	**Leu1490Leu**
**III**	**Heterozygous**	**4472C>T**	**Leu1491Phe**
III	Heterozygous	4584C>T	Pro1528Ser
III	Homozygous	4619A**>**T	Thr1540Ser
**III**	**Heterozygous**	**4670T>C**	**Leu1557Ser**

**Figure 2:**
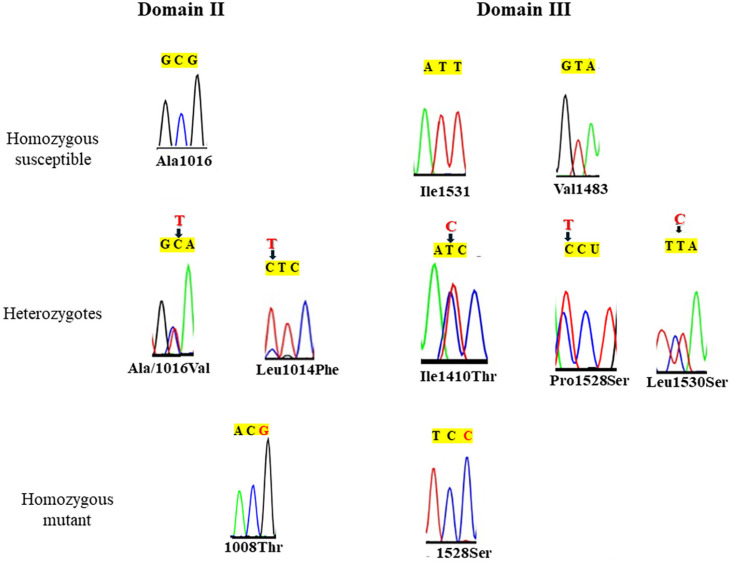
Chromatogram of mutations identified in partial segments of Domain II (480bp) and Domain III (740bp) of the sodium channel gene in *Ae. albopictus* populations. Red letters correspond to the substituted nucleotides.

In the IIS6 domain, susceptible alleles were identified at position 1016 (A1016: GCG, encoding the amino acid alanine). We also found the heterozygotes for the V1016A mutation at position 1016 (GTA to GCA, alanine to valine) associated with resistance to pyrethroid insecticides in *Musca domestica* ^18^. Finally, the homozygous mutant was identified at position 1008 (Thr1008: ACG), which codes for the amino acid threonine (Figure 2). In Domain III, the susceptible homozygote was found at position 1483 (V1483: GTA) coding for the amino acid valine/valine. Heterozygous alleles were found at position 1530 (L1530S: TTA to TCA), coding for the amino acid leucine to serine, as well as the mutation (I1410Thr: ATC to ACC) coding for the amino acid isoleucine to threonine; mutation 1528 (P1528S: CCU to TCU), coding for the amino acid proline to serine. The homozygous mutant 1528S (TCC), which codes for the amino acid serine to serine, was also found. Likewise, no kdr mutation was detected in domain IV.

## DISCUSSION

The Yucatan State is recognized as an endemic area for all four serotypes of the dengue virus^8^. The Asian tiger mosquito presents an additional potential hazard for the dissemination of flavivirus. The species *Ae. aegypti* and *Ae. albopictus* commonly coexist in household settings, leading to the application of pesticides targeting both species during vector control efforts^1,12^. Several populations of *Ae. aegypti* in Mexico have exhibited diminished sensitivity to pyrethroids, along with an elevated frequency of mutated genes in the sodium channel and detoxifying enzymes. Thus, the resistance expressed in *Ae. aegypti* to pyrethroids could be conditioned by mechanisms other than in *Ae. albopictus*, as the latter has only been recently introduced in the region^10,12,14,19^. In contrast, investigations conducted in Quintana Roo, Chiapas, and Yucatan have revealed that populations of *Ae. albopictus* remain susceptible to the pyrethroids permethrin and deltamethrin, as well as the organophosphates malathion and chlorpyrifos, and the carbamates bendiocarb and propoxur^11,14,15^. In this study, *Ae. albopictus* was susceptible to the insecticide lambda-cyalothrin. Notably, resistance monitoring is crucial for effective management. In 2015, *Ae. albopictus* populations collected in a neighborhood of Tapachula city (February 5), Mexico, exhibited a moderate level of resistance to permethrin. When the same population was re-evaluated the following year, they were found to have a susceptibility profile to the pyrethroid insecticide^11,12^. To the best of our understanding, the presence of Asian tiger mosquito populations resistant to the organophosphates chlorpyrifos, malathion, and temefos has been observed exclusively in Tapachula city, Chiapas State. This resistance has been established by detecting increased levels of acetylcholinesterase activity in the detoxification of insecticides in mosquitoes^12^. We found mosquitoes with heterozygous and homozygous mutant alleles, despite the fact that *Ae. albopictus* was susceptible to the pyrethroid lambda-cyalothrin. In Mexico, we reported for the first time a mutant allele with a T to C change at position 1410Thr (ATC/ACC) generating a heterozygotes in Domain III that codes for the aminoacid threonine. This mutation was previously identified in *Ae. albopictus* in rural populations of southern China and Rome city, Italy^20-22^. The early detection of mutant alleles in mosquitoes is essential for the development of insecticide resistance management strategies. Previously, *Ae. albopictus* in Tapachula city, Chiapas State, was screened for mutations (F1534) but none were identified^11^. We found mutant alleles in a rural population of the Asian mosquito. Therefore, more surveillance actions should be carried out in Yucatan to find the frequency and distribution of mutant alleles in sodium channel domains, in addition to describing the factors that can cause mutations in the sodium channel gene in mosquitoes and that modify the insecticide susceptibility profile. Mutations associated with resistance to pyrethroid insecticides detected in mosquitoes other than *Ae. albopictus* were also discovered in this research (Table 1). *Anopheles sinensis* and *Anopheles gambiae* have been shown to have Asp to Tyr and Leu to Ser, respectively^23,24^.

This study limitations include the lack of sensitive strains of *Ae. albopictus*. As a result, the bioassays carried out only examined the mortality rates of mosquitoes in insecticide-containing bottles in comparison to those in ethanol-containing control bottles.

## CONCLUSION

Synonymous and non-synonymous mutations were detected in *Ae. albopictus* mosquitoes obtained from a rural community. Consequently, the emergence of these mutations necessitates the implementation of integrated vector management strategies. This is imperative in order to effectively control mutations and prevent the dissemination and proliferation of resistant alleles within the region.
